# Identification, localization and expression of NHE isoforms in the alveolar epithelial cells

**DOI:** 10.1371/journal.pone.0239240

**Published:** 2021-04-21

**Authors:** Safa Kinaneh, Yara Knany, Emad E. Khoury, Reem Ismael-Badarneh, Shadi Hamoud, Gidon Berger, Zaid Abassi, Zaher S. Azzam

**Affiliations:** 1 Ruth & Bruce Rappaport Faculty of Medicine, Department of Physiology, Technion, Israel Institute of Technology, Haifa, Israel; 2 Youth Center for Science, Ort Braude College of Engineering, Karmiel, Israel; 3 Internal Medicine “E”, Rambam: Human Health Care Campus, Haifa, Israel; 4 Internal Medicine “B”, Rambam: Human Health Care Campus, Haifa, Israel; Max Delbruck Centrum fur Molekulare Medizin Berlin Buch, GERMANY

## Abstract

Na+/H+ exchangers (NHEs), encoded by Solute Carrier 9A (SLC9A) genes in human, are ubiquitous integral membrane ion transporters that mediate the electroneutral exchange of H+ with Na+ or K+. NHEs, found in the kidney and intestine, play a major role in the process of fluid reabsorption together via Na+,K+-ATPase pump and Na+ channels. Nevertheless, the expression pattern of NHE in the lung and its role in alveolar fluid homeostasis has not been addressed. Therefore, we aimed to examine the expression of NHE specific isoforms in alveolar epithelial cells (AECs), and assess their role in congestive heart failure (CHF). Three NHE isoforms were identified in AEC and A549 cell line, at the level of protein and mRNA; NHE1, NHE2 and mainly NHE8, the latter was shown to be localized in the apical membrane of AEC. Treating A549 cells with angiotensin (Ang) II for 3, 5 and 24 hours displayed a significant reduction in NHE8 protein abundance. Moreover, the abundance of NHE8 protein was downregulated in A549 cells that were treated overnight with Ang II. NHE8 abundance in whole lung lysate was increased in rats with 1-week CHF compared to sham operated rats. However, lower abundance of NHE8 was observed in 4-week CHF group. In conclusion, we herein show for the first time, the expression of a novel NHE isoform in AEC, namely NHE8. Notably, Ang II decreased NHE8 protein levels. Moreover, NHE8 was distinctly affected in CHF rats, probably depending on the severity of the heart failure.

## Introduction

Alveolar fluid clearance has been shown to be an important mechanism in keeping the airspaces free of edema in both cardiogenic and non-cardiogenic states [1,2]. There is a large body of evidence that the removal of alveolar fluid is attained by the alveolar epithelial active sodium transport; by which sodium passively enters the alveolar epithelial cells (AEC) via apical amiloride-sensitive Na^+^ channel (ENaC) or other Na^+^ channels and is pumped out of the cells by basolateral Na^+^, K^+^-ATPase, an energy consuming process. Following sodium transport, water is extruded from the alveolar airspaces [3–5]. It has been shown that the survival of acute lung injury patients, directly correlated with the rate of alveolar fluid clearance [6].

The sodium hydrogen exchanger (NHE) family includes several isoforms, which have different characteristics, including cell-compartment localization, plasma membrane distribution and organ-dependent function [7–9].

The evidence regarding NHE expression and role in the lungs, particularly in alveolar epithelial cells, is scarce. According to the evidence on NHEs in the kidney and intestine; water reabsorption is achieved by the function of Epithelial Na^+^ channel (ENaC), Na^+^,K^+^-ATPase pump along with Na^+^/H^+^ Exchangers (NHEs) [10,11]; thus, it is conceivable to assume that NHE may contribute to this transport in the lung, specifically in AEC. Notably, NHE in airway epithelium, like the mutation in NHE3 was identified as additional risk factors for airway infections in cystic fibrosis patient and loss of NHE function impaired mucociliary clearance [12,13]. Therefore, the major objective of this work is to address whether NHE isoforms are expressed in alveolar epithelial cells in healthy and congestive heart failure (CHF) rats. Then, to determine which isoforms are expressed and conceivably attempt to localize their site.

## Materials and methods

### Animals

Experiments were performed on adult male Sprague Dawley rats (N = 28) (Harlan Laboratories Ltd. Jerusalem, 275–350 g). Rats were provided water and food *ad libitum*. The use of animals in the present study was approved by the Technion Institutional Animal Care and Use Committee and it is according to NIH guidelines (Ethics # IL-054-05-15).

Under pentobarbital anesthesia (50 mg/kg, Intraperitoneal (IP)), congestive heart failure rat model was induced by surgically creating Aorto–Caval fistula (ACF) as previously described [14]. The abdomens of Sham controls were opened and sutured back again without creating an A–V fistula. After surgery, rats were placed in metabolic cages for 7 or 28 days. One or four weeks following surgery, rats were sacrificed (IP Pentobarbital, 60 mg/kg); lungs were collected and stored at -80° C. It is noteworthy that 3 rats did not survive the procedure of ACF.

### Alveolar epithelial type II cell isolation

Alveolar epithelial type II cells were isolated from the lungs of the various experimental groups based on a method described by Dobbs et al. [15]. Briefly, lungs were instilled and digested with elastase-containing solution (Worthington, Cat# LS002294). Following this, the lungs`lobes were chopped to release detached cells. Then, cells were plated on IgG (Sigma Aldrich, Cat# A9525) coated plates to get rid of contaminating macrophages. The non-adherent cells, mainly AEC, were recovered and plated for further experimentations. The purity of AEC was assessed by modified papanicolaou stain based on the presence of dark blue inclusions and ranged between 80–92%. Cell viability was assessed by trypan blue exclusion (>95%).

### Cell culture

AECII or A549 cells were cultured on plastic dishes in DMEM supplied with 10% FBS, penicillin (100 U/ml), streptomycin (0.1 mg/ml), gentamycin (0.05 mg/ml) amphotericin B (0.25 μg/ml), and L-Glutamine (2mM), and were incubated at 37° C with 5% CO2 in a humidified incubator. A549 cells were plated for 24 hours with medium-containing serum followed by 24-hour incubation with medium-free serum, then treated with Ang II (Cat# A9525, Sigma Aldrich).

### Preparation of basolateral membranes

Basolateral membrane (BLM) proteins (≈1.0 g net weight) were isolated from peripheral lung tissue (i.e., the distal 3 to 4 mm). 800–1000μl of homogenization buffer (300mM mannitol in 12 mM HEPES pH 7.4, 3 mM EGTA pH 8.0, 1 mM EDTA pH 8.0, 2 ug/ml leupeptin, 100 mM PMSF, 10 mg/ml TPCK) was added to the tissue. BLMs were isolated using a Percoll gradient (Sigma Aldrich, Ca# 77237) [16]. Briefly, tissue was homogenized, and centrifuged twice to discard the nuclear and mitochondrial pellet. Supernatant was centrifuged at 48,000 g for 30 minutes, and BLM fraction was recovered after the membrane pellet was centrifuged in a 16% Percoll gradient at 48,000 g for 30 minutes and then the ring of BLM was collected.

### Reverse transcriptase PCR (RT-PCR)

RNA was isolated from AECII and A549 cells using RNeasy Mini kit (Qiagen, Cat# 74104). While RNA purification of lung tissue was achieved using Tri-Reagent (Life Technologies, Cat# 15596026). All steps were done according to the manufacturer’s instructions. The isolated RNA was converted to cDNA using Maxima first strand cDNA kit (Thermo Scientific, Cat# K1671).

The primers used for RT-PCR are listed in Table 1. cDNA template (10-100ng), forward and reverse primers (0.4μM) and Taq PCR Master mix (Tiagen, Cat# KT201) were added all together to a final volume of 25 μl and placed in thermo cycler machine under the following thermal conditions: 95°C 5 min; 30 cycles of denaturing at 95°C for 30 sec, annealing at 58–62°C for 30 sec, and extension at 72°C for 1min; a final extension at 72°C for 5 min. Following the amplification procedure, PCR products were separated on 1.5% agarose gel with 100 bp DNA ladder and visualized with UV light.

**Table 1:** 

Gene	Species	Direction 5’ 3’	Primer sequence	Starting base	Amplicon size (bp)
NHE1-SLC9A1	Rattus norvegicus	Forward	TCTGCCGTCTCAACTGTCTCTA	2568	656
		Reverse	TACTGCCCTTTGGGGATGAAA	3223	
NHE2- SLC9A2	Rattus norvegicus	Forward	CAAGTTGCCCACGATTGTGC	492	709
		Reverse	GGCTGTGATCGCCATGATGC	1200	
NHE3- SLC9A3	Rattus norvegicus	Forward	TTGGCCAAAATCGTCTTCCAT	352	326
		Reverse	TTCAGTTCGCCCATCAGGCCA	677	
NHE8- SLC9A8	Rattus norvegicus	Forward	GTGTGTTTGCATTTCTTGGCCT	1094	532
		Reverse	AAGGGGTTCAGATACTTGGCAT	1625	
GAPDH	Rattus norvegicus	Forward	ATGCTGGTGCTGAGTATGTC	323	162
		Reverse	AGTTGTCATATTTCTCGTGG	484	
SP-C SFTPC	Rattus norvegicus	Forward	TATGACTACCAGCGGCTCCT	324	329
		Reverse	CTTTGCGGAGGGTCTTTCCT	648	
T1-α Pdpn	Rattus norvegicus	Forward	CCATCGGTGCGCTAGAAGAT	307	494
		Reverse	GGCAAGGTGGAAGCTCTCTT	800	
NHE1-SLC9A1	Homo sapiens	Forward	GTCATCAGCACCCTGCTCTT	2036	761
		Reverse	AGCATCTGGTTCCAGGCTTC	2796	
NHE2- SLC9A2	Homo sapiens	Forward	GTGTCTACCGTGGGCAAGAA	1453	758
		Reverse	TGTCTCTCACTTGTGTCGGC	2210	
NHE8- SLC9A8	Homo sapiens	Forward	TCTTGCCAGAGAGTGTTGCT	467	560
		Reverse	TGTTTGCCACCCACTGACAT	1026	

### Cell lysate and western blot analysis

Equal amounts of protein from lung homogenate, total AEC lysate, or Basolateral plasma membranes (BLMs), were resolved by 10% SDS-PAGE and analyzed by immunoblotting with specific antibodies against, NHE8 (Novus, Cat# NB110-62091) isoform and GAPDH (Santa Cruz, Cat# sc-25778), used as internal control.

### Immunofluorescence

A549 or AEC were fixed with 4% PFA then washed with DPBS, incubated with a blocking solution and later incubated overnight at 4° C with primary antibody against NHE8; subsequently, cells were incubated with secondary antibody Alexa-Fluor 594 Donkey anti mouse IgG (Life Technologies, Cat# A21203, 1/100 dilution), then washed, and finally mounted with Dapi Immunomount. Sections were visualized using Zeiss Axio observer inverted microscope system.

### Serum BNP

Serum BNP levels were measured by ELISA (AssayMax rBNP-32 ELISA kit, Assaypro, St. Charles, MO, USA). Serum BNP concentration of < 100 pg/ml was considered normal.

### Statistical analysis

Data were presented as mean ± SEM; n is the number of animals in each study group. One way analysis of variance was used when multiple comparisons are made followed by a multiple comparison test (Tukey) when the F statistic indicated significance. To analyze paired data, we used unpaired t-test to assess the differences between the study groups. Results were considered significant when p < 0.05.

The Kolmogorov-Smirnov test was used to analyze the normality of the groups. The Levene’s test was used for comparing the equality of variances. The Student t-test for independent groups was used to compare between two study groups. Two-tailed p value of 0.05 or less was considered to be statistically significant.

## Results

### Identification of Na^+^/H^+^ exchanger isoforms expressed in alveolar epithelial and A549 cells

**Fig 1:**
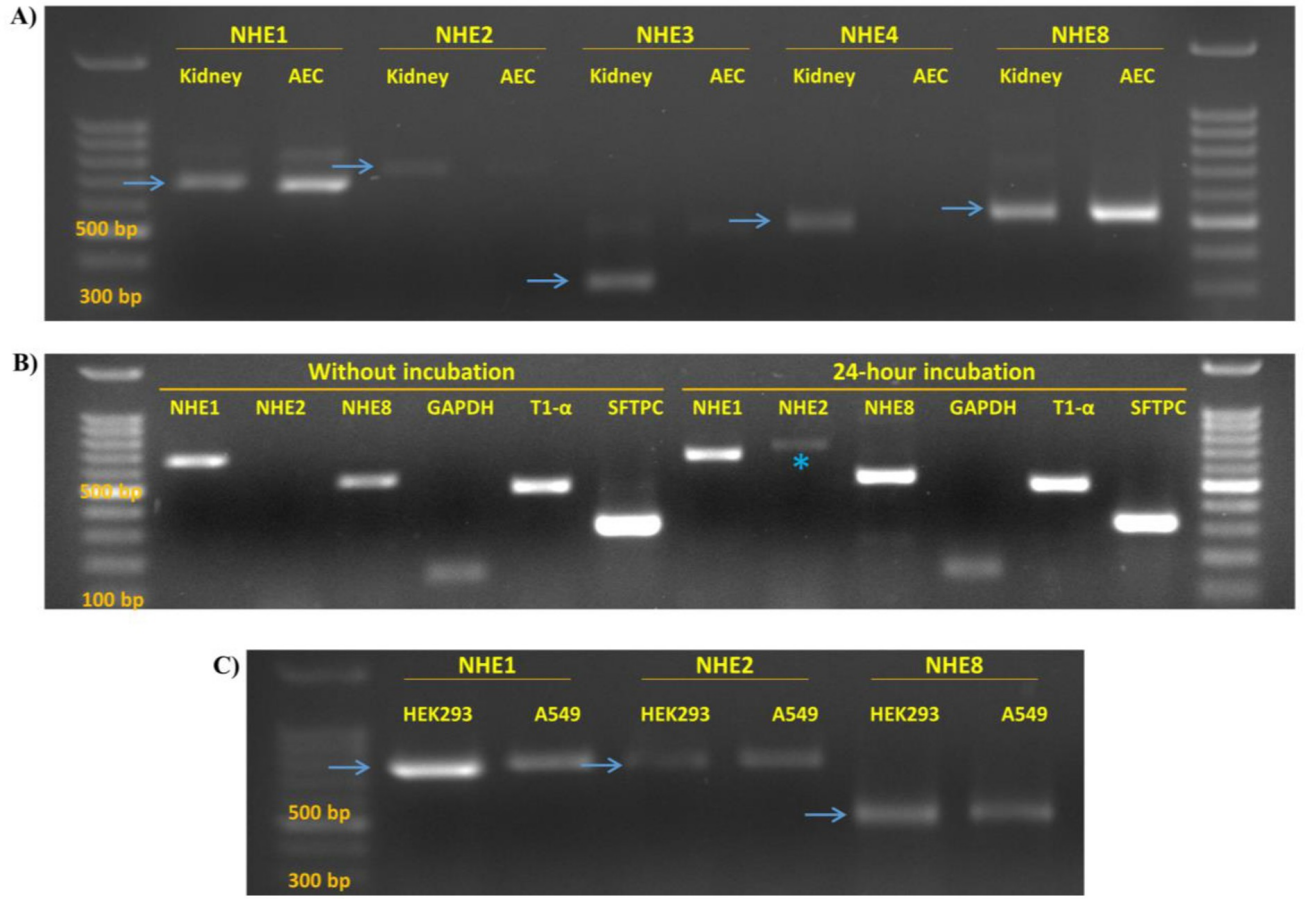
(A) RT-PCR showing the expression of NHE isoforms in isolated AEC as compared to kidney sample. Positive signals of NHE1 and NHE8, but not NHE2 and NHE3, were detected in AEC. (B) RT-PCR that illustrates NHE isoforms expression in AEC that were/or not 24-hour incubated. The asterisk indicate NHE2 expressed in AEC following incubation, but was absent when AEC were freshly assessed. (C) RT-PCR demonstrating the expression of NHE1, NHE2 and NHE8 in A549 and Human embryonic kidney 293 cell line (HEK293) that served as positive control. The blue arrows point to the expected product length. AEC—alveolar epithelial cells. NHE—Na^+^/H^+^ Exchanger. GAPDH—Glyceraldehyde 3- phosphate dehydrogenase. T1-α–AECI marker, SFTPC—Surfactant Protein C, AECII marker.

Our major focus was on NHE isoforms that are primarily localized to cell membranes, thus can potentially contribute to the alveolar active sodium transport, and eventually alveolar fluid clearance. Among these are NHE1-5 and NHE8. NHE5, however, was not included in our experiments as it is reported to be exclusively expressed in the brain [17,18]. By using RT-PCR and targeted primers to each isoform, the expression of NHE1 and NHE8 was confirmed in both cell types of isolated AEC (Fig 1A). Moreover, NHE2 was found to be expressed only following cells incubation for 24 hours (Fig 1B); this observation might be attributed to the differentiation of AEC type II into type I. Similarly, these exchangers, namely NHE1, NHE2 and NHE8 are expressed in A549 cell line, known to have characteristic features of AECII (Fig 1C). Surprisingly, NHE3 expression was not demonstrated in neither cell types, whereas the expression of unexpected novel isoform, namely NHE8, was established.

**Fig 2:**
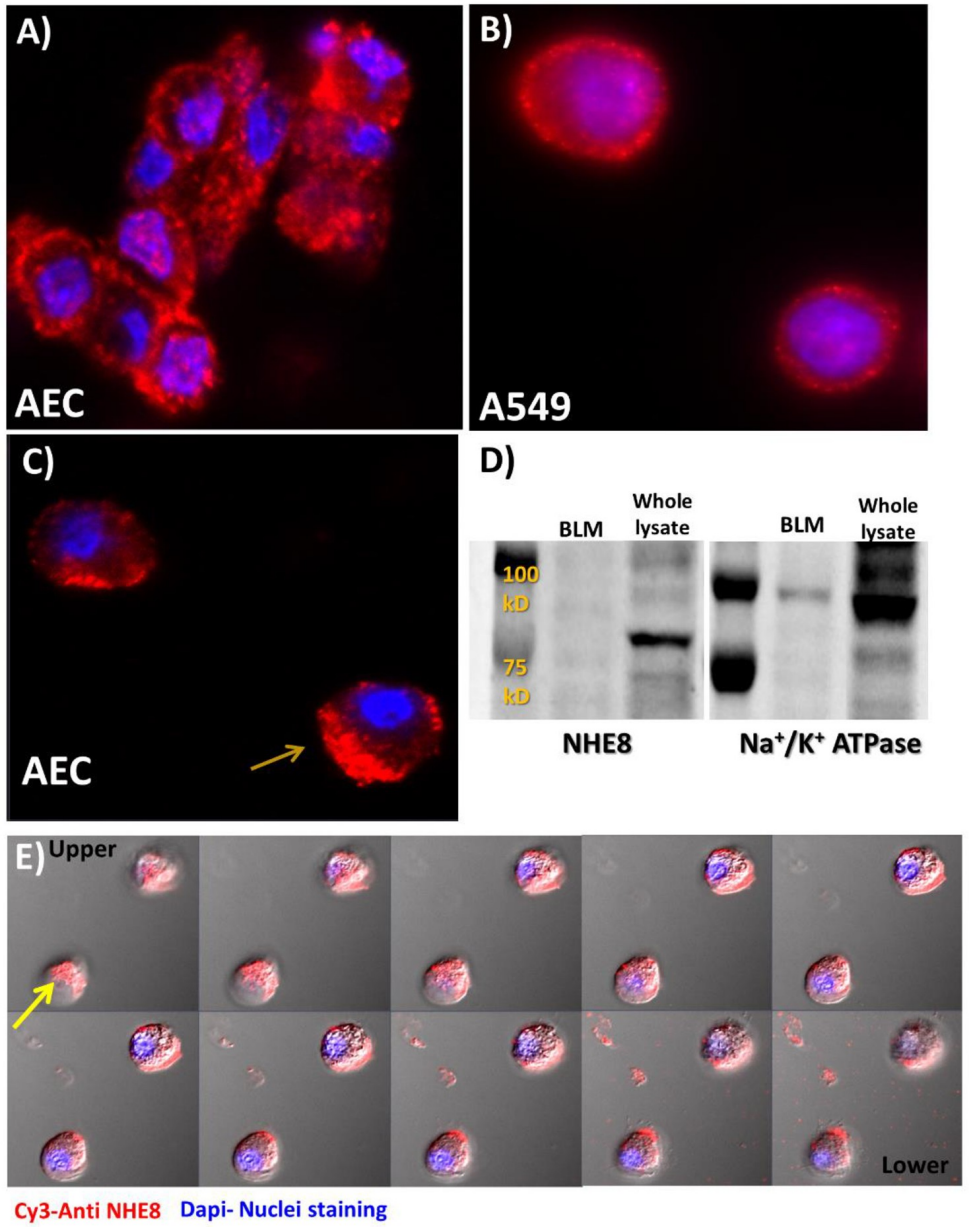
Immunofluorescence staining of NHE8 stained with Cy3 (Red) in isolated AEC (A and C) and A549 cell line (B), showing strong staining at the plasma membrane and a polar distribution in part of the cells (C). Nuclei were stained with Dapi (blue). (D) Western blot to BLM and whole lung lysate showing no existence of NHE8 in BLM fraction; yet, Na^+^/K^+^ ATPase used as a marker of basolateral membranes do exist. (E) NHE8 localization to apical membrane of alveolar epithelial cells was achieved by series of images taken by confocal microscope. The first image refers to the apical side while the last one refers to the basolateral side. The red signal got weaker as we imaged deeper; more obvious in the cell pointed with yellow arrow. BLM—basolateral membranes. NHE—Na+/H+ Exchanger.

Based on previous reports, NHE8 might be localized to intracellular compartments or to the plasma membrane [19,20]. Immunofluorescence staining to NHE8 in AEC was positive in both compartments. Whereas, in A549 cells, the staining was exclusively confirmed in the plasma (Fig 2A–2C).

To determine whether NHE8 was localized to the apical or basolateral membrane, we used confocal microscope and collected sets of images at different depths. As illustrated in Fig 2E, the deeper we imaged the weaker the red signal we got and the closer we were to the basolateral side, which indicated apical localization of NHE8. Then we needed to validate this observation by isolating apical membranes of AEC; however, unfortunately, we did not manage to do that due to technical limitations. Notably, NHE8 protein was not detected in BLM of lung fractions, whereas Na+, K+-ATPase is normally localized (Fig 2D).

### NHE8 levels are decreased in A549 cells following treatment with Angiotensin II

**Fig 3:**
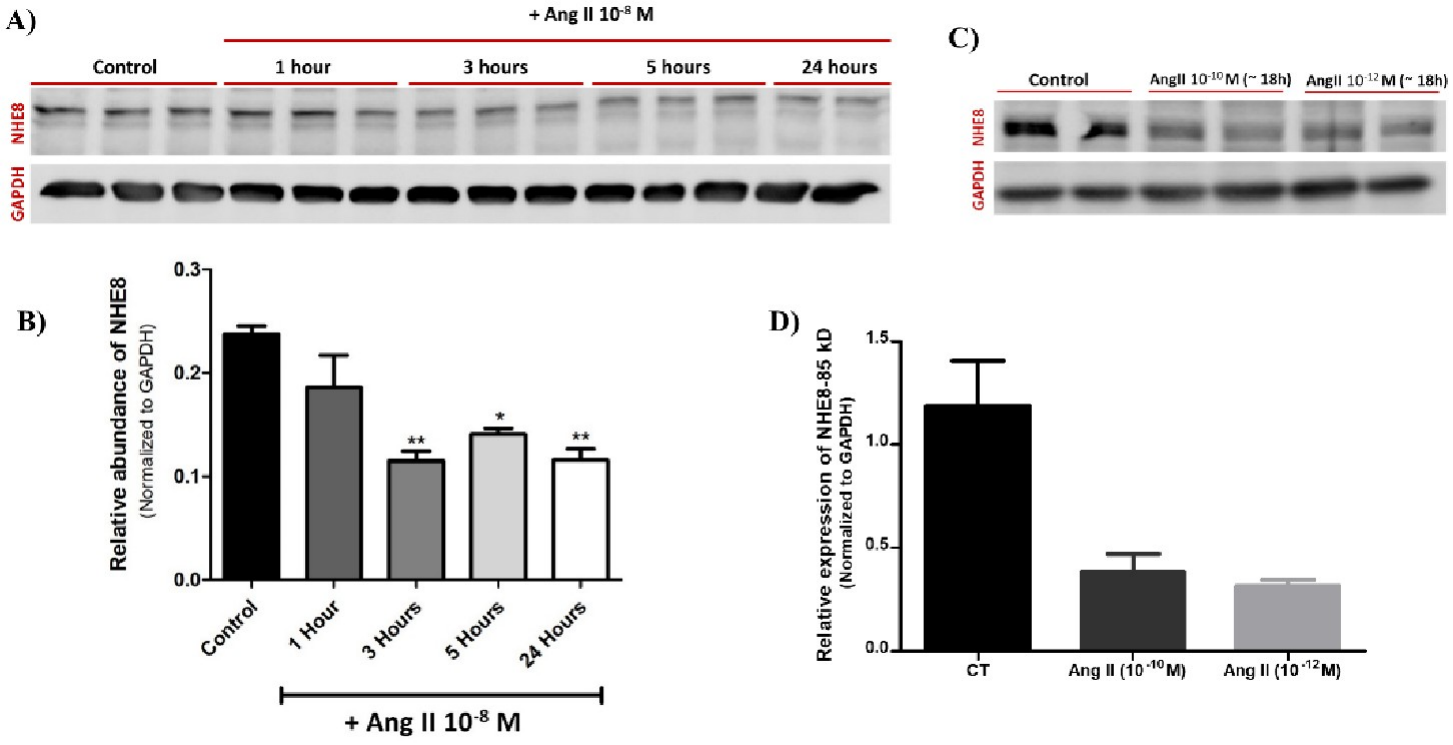
Effect of angiotensin II treatment on NHE8 protein abundance in A549 cells. A concentration of 10^−8^ M of Ang II was added to A549 cells medium for 1, 3, 5, and 24 hours. NHE8 protein abundance, as assessed by western blotting, significantly was decreased following Ang II treatment for 3, 5 (N-3 each) & 24 hours (N-2) (Figures A & B). * P value <0.05 as compared to control. ** P value < 0.01 as compared to control. Notably, the decrease in NHE8 1 hour (N-3) following the administration of Ang II to A549 did not reach clinical significance. A549 cells were treated overnight (~ 18h) with Ang II at two concentrations (10^−10^ M and 10^−12^ M, n = 2 in each), which resulted in decreased NHE8 protein levels as shown by western blot (C) and the relevant quantification after normalizing to GAPDH (D). Ang II—Angiotensin II. CT—Control. NHE—Na+/H+ Exchanger. GAPDH—Glyceraldehyde 3- phosphate dehydrogenase. The bars represent mean ± SEM.

Recently, we have shown that angiotensin II (Ang II) impaired the ability of the lungs to clear edema by downregulating alveolar active Na^+^ transport [21]. Notably, there are no known inhibitors or activators of NHE and assuming that NHE8 may contribute to fluid clearance; we investigated whether it is affected by Ang II. For this purpose, we examined the Ang II effect on NHE8 protein expression in a time -dependent manner. We treated A549 cells with Ang II (10^−8^ M) for 1, 3, 5 and 24 hours. As depicted in Fig 3A & 3B, NHE8 protein abundance, as assessed by western blot analysis, was significantly downregulated following Ang II treatment for 3, 5 (N-3 each) & 24 hours (N-2) (Fig 3A & 3B) as compared to control (N-3). Notably, 1 hour (N-3) following the administration of Ang II to A549; NHE8 was also decreased; however, it did not reach clinical significance.

When A549 cells were treated overnight (~18h) with Ang II at concentrations of 10^−10^ M and 10^−12^ M; NHE8 abundance was downregulated in both concentrations as compared to untreated cells (Fig 3C and 3D). Notably, this decrease did not reach statistical significance due to the small number of samples in each group (N = 2).

### NHE8 protein abundance in lung tissue of CHF-operated rats

**Fig 4:**
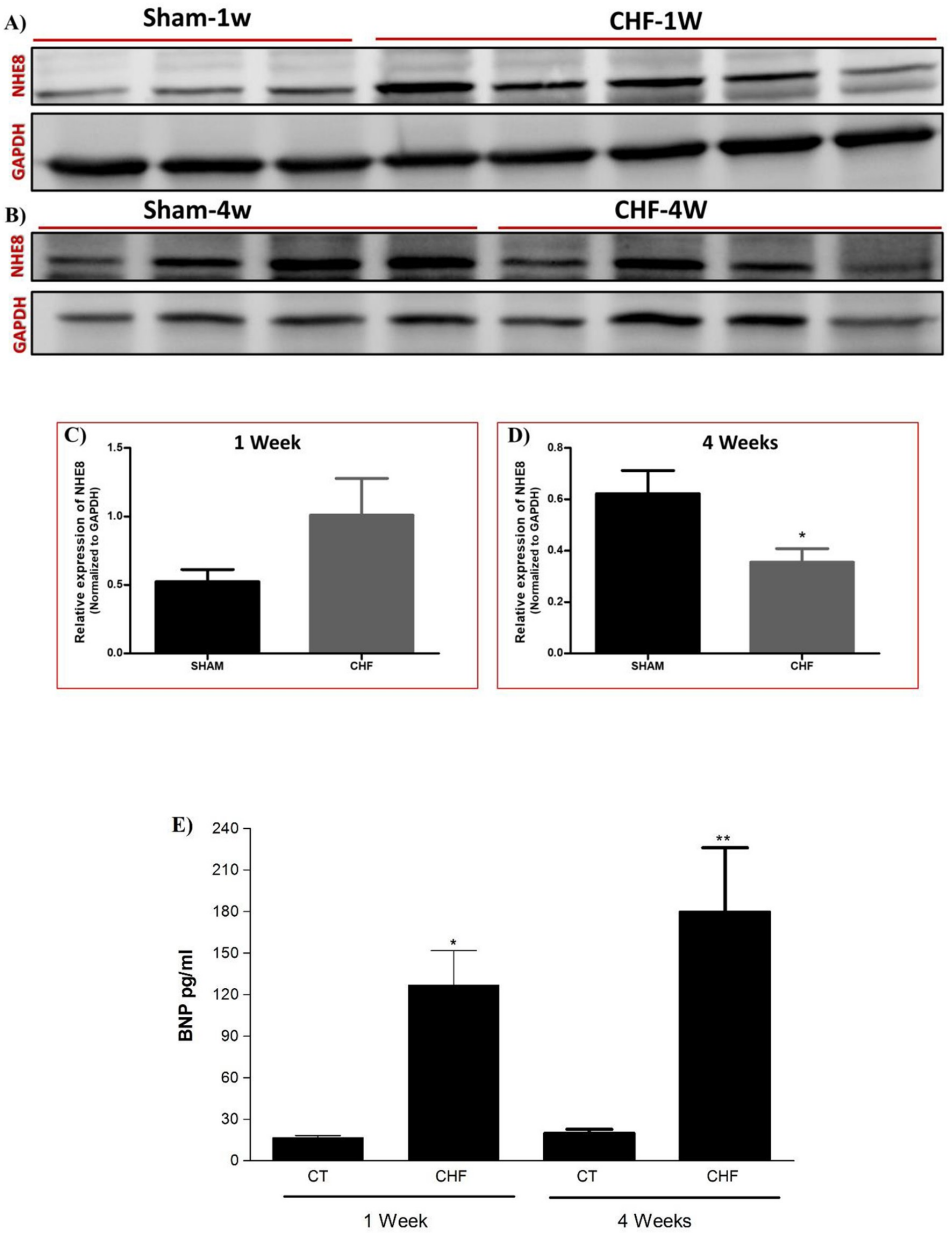
NHE8 immunoreactive levels in the lungs of CHF-1w and CHF-4w rats. (A) Western blot showing increased NHE8 protein levels in CHF-1w (n = 5) as compared to their Sham-1w (n = 3). (C) Quantification of NHE8; normalized to GAPDH. (B) Western blot showing decreased NHE8 protein levels in CHF-4w (n = 4) as compared to their Sham-4w (n = 4). (D) Quantification of NHE8; normalized to GAPDH. *P_Value <0.05. (D) The severity of CHF was noted by the levels of BNP. serum BNP levels was significantly increased in the 1—week CHF group as compared to control group; *P = 0.016. Similarly in the 4 –weeks CHF group, BNP was significantly increased as compared to control; **P < 0.01. The bars represent mean ± SEM. CHF—Congestive Heart Failure. NHE—Na+/H+ Exchanger. GAPDH—Glyceraldehyde 3- Phosphate Dehydrogenase. Serum BNP of < 100 pg/ml was considered normal.

Based on the up mentioned finding of NHE8 abundance in AEC, and its plasma membrane occurring profile, we assume that NHE8 has an important role in alveolar fluid clearance; therefore, we were interested to explore whether its expression is modified in a model of congestive heart failure (CHF) as compared to sham rats. We performed western blot to evaluate NHE8 protein expression in lungs, one (CHF-1w) and four (CHF-4w) weeks following ACF procedure, as compared to sham operated rats. CHF-1w rats exhibited an increased NHE8 protein abundance compared to sham-1w rats, yet this elevation was not significant, possibly due to high variability of edema severity among CHF rats (Fig 4A and 4C). However, NHE8 immunoreactive levels were significantly decreased in CHF-4w rats as compared to sham 4-w rats (Fig 4B and 4D). This distinct expression suggests that NHE8 expression may be related to the severity and progression of CHF. It is worth mentioning that the severity of CHF was noted by measuring BNP levels in the serum of the various CHF and control groups. As depicted in Fig 4E, BNP levels was increased by more than 7 folds of in the 1—week CHF group as compared to control group; P = 0.016. Noteworthy, in the 4 –weeks CHF group, BNP was increased by 9-fold as compared to control; P < 0.001.

## Discussion

Keeping alveolar fluid spaces free of edema is essential for efficient gas exchange.

The molecular basis underlying alveolar fluid clearance (AFC) process involves active sodium transport from the apical to the basolateral side of the alveolar epithelium, generating an osmotic gradient that drives water movement and clearance. Mainly, ENaC and Na^+^, K^+^-ATPase are considered to be the main players in this process [3,22,23].

Alveolar fluid clearance process is equivalent to the kidney in terms of water and sodium reabsorption. Yet, in the kidney, another participant, alongside with ENaC and Na^+^, K^+^-ATPase, is involved; that is NHE3. It accounts for most of the sodium reabsorption in the proximal tubules [24]. However, very little is known regarding NHE expression in the lung and particularly in alveolar epithelial cells. Since ENaC contributes to only 40–60% of sodium transport in most species [25], the rest of the sodium entry must be driven by other pathways. Based on this evidence, we were intrigued to investigate the potential role of NHEs, their expression pattern in alveolar epithelial cells, and to evaluate their involvement in pulmonary edema pathology and AFC process. We hypothesized that at least one NHE isoform might be expressed in alveolar epithelial cells that may contribute to vectorial sodium movement.

NHE is a large family containing about nine distinct isoforms. NHE3 has distinctive characteristics and is directly involved in water reabsorption in the kidney, as mentioned previously [11,24]. Thus, we first, investigated its expression in AEC; however, there was no evidence for NHE3 expression in isolated AEC; an observation that was supported by RT-PCR experiments (Fig 1A). Therefore, other NHE isoforms known to be localized to the plasma membrane were investigated. Based on previous reports [26,27]; four NHE isoforms were studied; NHE-1, 2, 4, and 8. Our study demonstrated the expression of NHE1 and NHE8 in AEC (Fig 1A), whereas NHE2 and NHE4 expression was not evident.

Notably, while manifesting AECII differentiation properties, the isolated cells were divided into two groups; a freshly processed group and a 24-hour incubation group. NHE isoforms analysis with RT-PCR, demonstrated the expression of NHE2 only in the incubated cells (Fig 1B). Conceivably, this observation may be related to the assumption that NHE2 is localized in AECI.

The AECII cell-like cell line, A549, was used to validate NHE isoforms expression pattern previously described in isolated AEC. Using RT-PCR, NHE1, NHE8 and NHE2 expression was observed in A549 cells as well (Fig 1C). This set of experiments demonstrated, to our knowledge, for the first time, that NHE8 is expressed in AEC. It is noteworthy that, its expression was confirmed in the kidney and intestine. Obviously, NHE8 expression is remarkably regulated especially in the neonatal intestinal brush border; while in the adults it is replaced with NHE3 [28,29]. Xu et al. demonstrated the apical localization of NHE-8 protein in rat intestinal epithelial cells [19]. Similarly, Goyal et al. have shown that NHE8 protein was expressed in apical membranes of the renal cortex [30]. Based on these intestinal and renal findings, we decided to focus on NHE8 and further investigate its expression pattern, localization within the cells and involvement in the pathogenesis of pulmonary edema. Therefore, a set of experiments was performed to characterize the localization and regulation of NHE8. Immunofluorescence staining of NHE8 displayed its presence in the plasma membrane of AEC and A549 cells, with indication to polarized abundance (Fig 2A–2C). Western blotting to isolated basolateral membranes of AEC was negative for NHE8 expression (Fig 2D). While, confocal microscope findings support the apical localization of NHE8 (Fig 2E). Therefore, it is conceivable to assume that NHE8 is localized on the apical side of AEC.

Unfortunately, there is no known selective inhibitor for NHE8 since it is not much studied and only recently has been discovered. Therefore, we decided to bypass this obstacle and study the response of this isoform to hormones and factors known to impair the ability of the lungs to clear edema.

Angiotensin II, the major component of renin-angiotensin aldosterone system (RAAS), has an important role in the regulation of arterial pressure, salt balance and body fluid volume homeostasis. Furthermore, its levels are increased in heart failure [31]. Recently, our group has shown that Ang II impaired AFC, partly by down-regulating Na^+^, K^+^-ATPase protein levels [21]. Assuming that NHE8 participates in vectorial sodium transport, we hypothesized that Ang II may affect NHE8 protein expression. Therefore, we conducted an experiment in which A549 cells were treated with Ang II (10^−8^ M) and examined NHE8 protein changes over different periods of time- 1, 3, 5 and 24 hours. As shown in Fig 3A & 3B, Ang II significantly decreased the levels of NHE8 mature protein at 3 (N = 3), 5 hours (N = 3) and 24 hours (N = 2) of treatment as compared to control (N = 3); it is noteworthy that NHE levels were decreased one hour (N-3) following the administration of Angiotensin, however, it did not reach statistical significance. Another set of experiments, included overnight treatment of A549 with Ang II at concentrations of 10^−10^ M and 10^−12^ M, showed decreased NHE8 abundance as compared to untreated cells (Fig 3C and 3D). Based on these findings, we concluded that Ang II downregulated NHE8 protein abundance in a dose- and time-dependent manner.

Notably, since Ang II treatment experiments involved cell incubation for up to 24 hours which might trigger AECI differentiation and in order to minimize the number of sacrificed animals, we favored with the use of A549 cells in this set of experiments, and not isolated AEC.

Furthermore, we investigated NHE8 expression in CHF rats, using ACF rat model. Rats were sacrificed 1 week (CHF-1w) or 4 weeks (CHF-4w) following ACF procedure. NHE8 was distinctly expressed in CHF rat lungs, in which CHF-1w NHE8 levels were increased, while in CHF-4w, NHE8 levels were decreased, as compared to sham-rats (Fig 4). This distinct expression pattern of NHE8 might suggest a protective role of NHE8 in CHF-1w that is driven by the need to clear excessive lung fluids; while the decreased levels in CHF-4w, might be a result of CHF severe condition, were even protective pathways are badly damaged.

The main limitation of this study was our inability to directly address NHE8 effect on AFC due to the lack of specific inhibitors or NHE8 knock-out mice models.

Acute lung injury and deterioration to ARDS is the manifestation of the recently erupted global pandemic, the novel coronavirus SARS-CoV-2 (COVID-19 disease). Therefore, potential involvement of NHEs in general and NHE8 in particular in the pathogenesis of SARS-CoV-2 induced ARDS, worth further evaluation and may contribute to the development of new therapeutic modalities [32,33].

**Fig 5:**
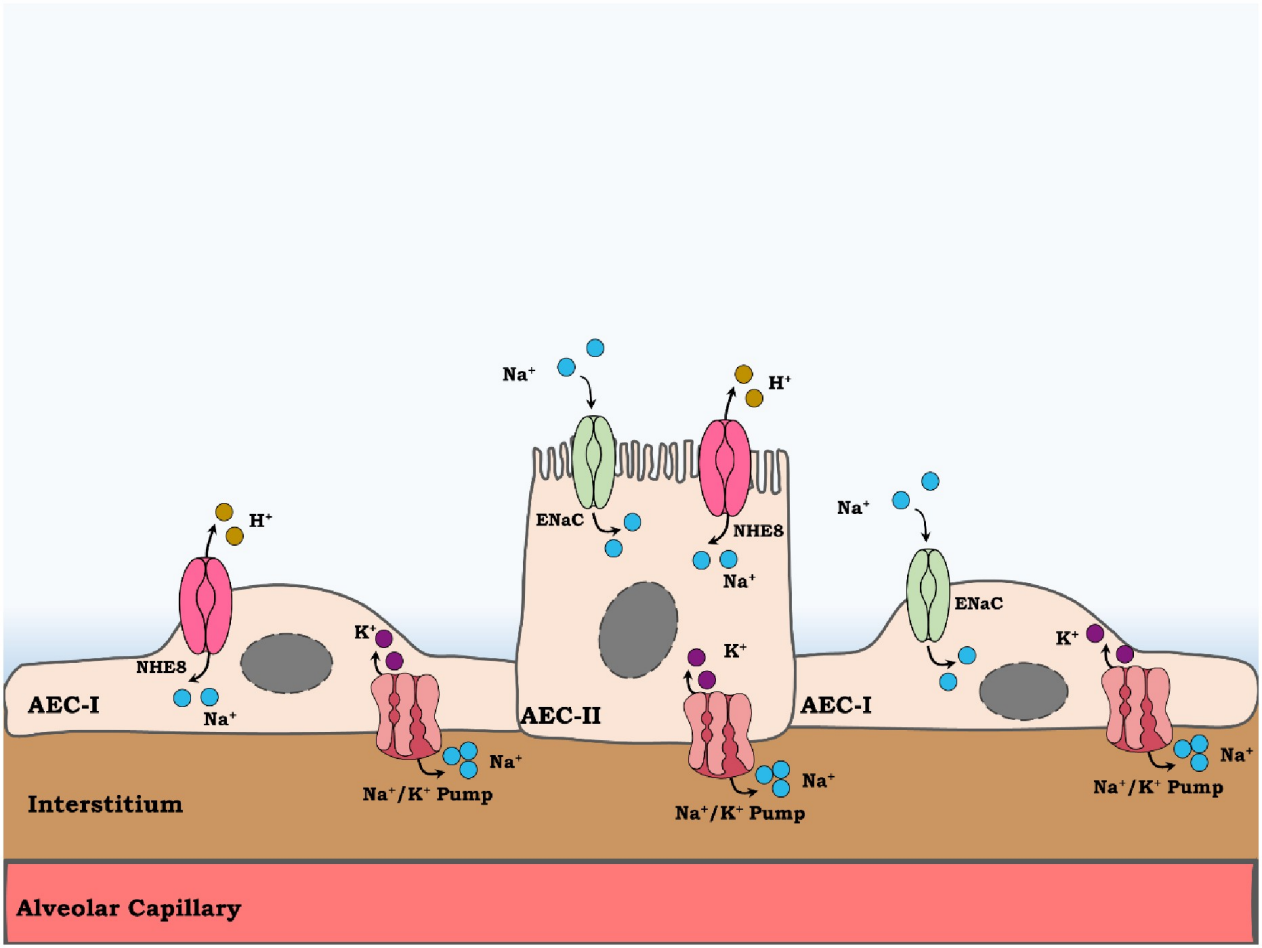
A schematic illustration of NHE8 role in sodium vectorial transport. NHE8 may be located to Alveolar epithelial cells type I (AECI) and II (ACEII), where in the latter it is localized to the apical membrane. By exchanging extracellular Na^+^ with intracellular H^+^, NHE8 contribute to Na^+^ vectorial transport along with epithelial sodium channel (ENaC). The entered sodium then is pumped out of alveolar cells by Na^+^, K^+^ ATpase pump. Sodium transport from alveolar space to the interstitium is accompanied by water movement and so edema clearance.

In summary, herein we report, for the first time, the presence of the NHE8 isoform in alveolar epithelial cells. It is localized in the apical membrane of AEC with consequent speculative contribution to ENaC function and vectorial sodium transport in alveolar epithelial cells. We also showed that Ang II regulates NHE8 protein abundance in a dose- and time- dependent manner, and that NHE8 protein levels are distinctly regulated in CHF-rats (Fig 5).

## Supporting information

S1 Checklist The ARRIVE guidelines 2.0: Author checklist. (PDF)

S1 Raw images (PDF)

## References

[1] Matthay MA. Resolution of pulmonary edema thirty years of progress. Am J Respir Crit Care Med. 2014;189: 1301–1308. 24881936 10.1164/rccm.201403-0535OE PMC4098087

[2] Hochberg I, Abassi Z, Azzam ZS. Patterns of alveolar fluid clearance in heart failure. Int J Cardiol. 2008;130: 125–130. 10.1016/j.ijcard.2008.03.015 18579236

[3] Vadasz I, Raviv S, Sznajder JI. Alveolar epithelium and Na,K-ATPase in acute lung injury. Intensive Care Med. 2007;33: 1243–1251. Available: http://www.ncbi.nlm.nih.gov/entrez/query.fcgi?cmd=Retrieve&db=PubMed&dopt=Citation&list_uids=17530222. 10.1007/s00134-007-0661-8 17530222 PMC7095466

[4] Sznajder JI, Factor P, Ingbar DH. Invited review: lung edema clearance: role of Na(+)-K(+)-ATPase. J Appl Physiol. 2002;93: 1860–6. Available: http://www.ncbi.nlm.nih.gov/htbin-post/Entrez/query?db=m&form=6&dopt=r&uid=12381775. 10.1152/japplphysiol.00022.2002 12381775

[5] Berthiaume Y, Folkesson HG, Matthay MA. Lung Edema Clearance: 20 Years of Progress Invited Review: Alveolar edema fluid clearance in the injured lung. J Appl Physiol. 2002;93: 2207–2213. 10.1152/japplphysiol.01201.2001 12433940

[6] Ware LB, Matthay MA. Alveolar fluid clearance is impaired in the majority of patients with acute lung injury and the acute respiratory distress syndrome. Am J Respir Crit Care Med. 2001;163: 1376–1383. Available: http://www.ncbi.nlm.nih.gov/entrez/query.fcgi?cmd=Retrieve&db=PubMed&dopt=Citation&list_uids=11371404. 10.1164/ajrccm.163.6.2004035 11371404

[7] Fliegel L, Frohlich O. The Na+/H+ exchanger: An update on structure, regulation and cardiac physiology. Biochem J. 1993;296: 273–285. 10.1042/bj2960273 8257412 PMC1137689

[8] Fliegel L. Regulation of myocardial Na+/H+ exchanger activity. Basic Res Cardiol. 2001;96: 301–305. 10.1007/s003950170036 11518184

[9] Sakuta H, Lin CH, Hiyama TY, Matsuda T, Yamaguchi K, Shigenobu S, et al. SLC9A4 in the organum vasculosum of the lamina terminalis is a [Na+] sensor for the control of water intake. Pflugers Arch Eur J Physiol. 2020;472: 609–624. 10.1007/s00424-020-02389-y 32372285

[10] Slepkov ER, Rainey JK, Sykes BD, Fliegel L. Structural and functional analysis of the Na+/H+ exchanger. Biochem J. 2007;401: 623–633. 10.1042/BJ20061062 17209804 PMC1770851

[11] Fenton RA, Poulsen SB, de la Mora Chavez S, Soleimani M, Dominguez Rieg JA, Rieg T. Renal tubular NHE3 is required in the maintenance of water and sodium chloride homeostasis. Kidney Int. 2017;92: 397–414. 10.1016/j.kint.2017.02.001 28385297 PMC5511580

[12] Pereira SVN, Ribeiro JD, Bertuzzo CS, Marson FAL. Association of clinical severity of cystic fibrosis with variants in the SLC gene family (SLC6A14, SLC26A9, SLC11A1 and SLC9A3). Gene. 2017;629: 117–126. 10.1016/j.gene.2017.07.068 28756021

[13] Sun DI, Tasca A, Haas M, Baltazar G, Harland RM, Finkbeiner WE, et al. Na + /H + exchangers are required for the development and function of vertebrate mucociliary Epithelia. Cells Tissues Organs. 2019. 10.1159/000492973 30300884 PMC6397095

[14] Khoury EE, Kinaneh S, Aronson D, Amir O, Ghanim D, Volinsky N, et al. Natriuretic peptides system in the pulmonary tissue of rats with heart failure: Potential involvement in lung edema and inflammation. Oncotarget. 2018;9: 21715–21730. 10.18632/oncotarget.24922 29774097 PMC5955134

[15] Dobbs LG. Isolation and culture of alveolar type II cells. Am J Physiol. 1990;258: L134–47. Available: http://www.ncbi.nlm.nih.gov/entrez/query.fcgi?cmd=Retrieve&db=PubMed&dopt=Citation&list_uids=2185652. 10.1152/ajplung.1990.258.4.L134 2185652

[16] Azzam ZS, Adir Y, Welch L, Chen J, Winaver J, Factor P, et al. Alveolar fluid reabsorption is increased in rats with compensated heart failure. Am J Physiol Lung Cell Mol Physiol. 2006;291: L1094–100. Available: http://www.ncbi.nlm.nih.gov/entrez/query.fcgi?cmd=Retrieve&db=PubMed&dopt=Citation&list_uids=16815890. 10.1152/ajplung.00180.2005 16815890

[17] Kurata T, Rajendran V, Fan S, Ohta T, Numata M, Fushida S. NHE5 regulates growth factor signaling, integrin trafficking, and degradation in glioma cells. Clin Exp Metastasis. 2019;36: 527–538. 10.1007/s10585-019-10001-6 31595389 PMC6834540

[18] Baird NR, Orlowski J, Szabó EZ, Zaun HC, Schultheis PJ, Menon AG, et al. Molecular cloning, genomic organization, and functional expression of Na+/H+ exchanger isoform 5 (NHE5) from human brain. J Biol Chem. 1999;274: 4377–4382. 10.1074/jbc.274.7.4377 9933641

[19] Xu H, Chen R, Ghishan FK. Subcloning, localization, and expression of the rat intestinal sodium-hydrogen exchanger isoform 8. Am J Physiol—Gastrointest Liver Physiol. 2005. 10.1152/ajpgi.00552.2004 15731506

[20] Wiebe SA, Plain A, Pan W, O’neill D, Braam B, Alexander RT. NHE8 attenuates Ca2+ influx into NRK cells and the proximal tubule epithelium. Am J Physiol—Ren Physiol. 2019. 10.1152/ajprenal.00329.2018 31042050

[21] Ismael-Badarneh R, Guetta J, Klorin G, Berger G, Abu-Saleh N, Abassi Z, et al. The role of Angiotensin II and cyclic AMP in alveolar active sodium transport. Bader M, editor. PLoS One. 2015;10: 1–13. 10.1371/journal.pone.0134175 26230832 PMC4521808

[22] Matalon S, Hardiman KM, Jain L, Eaton DC, Kotlikoff M, Eu JP, et al. Regulation of ion channel structure and function by reactive oxygen-nitrogen species. Am J Physiol Lung Cell Mol Physiol. 2003;285: L1184–9. Available: http://www.ncbi.nlm.nih.gov/entrez/query.fcgi?cmd=Retrieve&db=PubMed&dopt=Citation&list_uids=14604848. 10.1152/ajplung.00281.2003 14604848

[23] Matthay MA, Folkesson HG, Clerici C. Lung epithelial fluid transport and the resolution of pulmonary edema. Physiol Rev. 2002;82: 569–600. Available: http://www.ncbi.nlm.nih.gov/entrez/query.fcgi?cmd=Retrieve&db=PubMed&dopt=Citation&list_uids=12087129. 10.1152/physrev.00003.2002 12087129

[24] Vallon V, Schwark JR, Richter K, Hropot M. Role of Na+/H+ exchanger NHE3 in nephron function: Micropuncture studies with S3226, an inhibitor of NHE3. Am J Physiol—Ren Physiol. 2000;278: F375–F379. 10.1152/ajprenal.2000.278.3.F375 10710541

[25] Mutlu GM, Sznajder JI. Mechanisms of pulmonary edema clearance. Am J Physiol Lung Cell Mol Physiol. 2005;289: L685–95. Available: http://www.ncbi.nlm.nih.gov/entrez/query.fcgi?cmd=Retrieve&db=PubMed&dopt=Citation&list_uids=16214819. 10.1152/ajplung.00247.2005 16214819

[26] Brett CL, Donowitz M, Rao R. Evolutionary origins of eukaryotic sodium/proton exchangers. Am J Physiol—Cell Physiol. 2005;288: C223–C239. 10.1152/ajpcell.00360.2004 15643048

[27] Donowitz M, Ming Tse C, Fuster D. SLC9/NHE gene family, a plasma membrane and organellar family of Na +/H+ exchangers. Mol Aspects Med. 2013;34: 236–251. 10.1016/j.mam.2012.05.001 23506868 PMC3724465

[28] Gurney MA, Laubitz D, Ghishan FK, Kiela PR. Pathophysiology of Intestinal Na+/H+ Exchange. CMGH. 2017;3: 27–4. 10.1016/j.jcmgh.2016.09.010 28090568 PMC5235326

[29] Becker AM, Zhang J, Goyal S, Dwarakanath V, Aronson PS, Moe OW, et al. Ontogeny of NHE8 in the rat proximal tubule. Am J Physiol—Ren Physiol. 2007;293: F255–F261. 10.1152/ajprenal.00400.2006 17429030 PMC4119019

[30] Goyal S, Vanden Heuyel G, Aronson PS. Renal expression of novel Na+/H+ exchanger isoform NHE8. Am J Physiol—Ren Physiol. 2003;284. 10.1152/ajprenal.00352.2002 12409279

[31] Packer M. The neurohormonal hypothesis: a theory to explain the mechanism of disease progression in heart failure. J Am Coll Cardiol. 1992;20: 248–254. Available: http://www.ncbi.nlm.nih.gov/entrez/query.fcgi?cmd=Retrieve&db=PubMed&dopt=Citation&list_uids=1351488. 10.1016/0735-1097(92)90167-l 1351488

[32] Wu F, Zhao S, Yu B, Chen YM, Wang W, Song ZG, et al. A new coronavirus associated with human respiratory disease in China. Nature. 2020;579: 265–269. 10.1038/s41586-020-2008-3 32015508 PMC7094943

[33] Singer BD, Jain M, Budinger GRS, Wunderink RG. A Call for Rational Intensive Care in the Era of COVID-19. Am J Respir Cell Mol Biol. 2020;63: 132–133. 10.1165/rcmb.2020-0151LE 32315548 PMC7328253

