# Supplementary material for: Identification, localization and expression of NHE isoforms in the alveolar epithelial cells
Source: PLoS One. 2021 Apr 21;16(4):e0239240. doi: 10.1371/journal.pone.0239240 (PMC8059851; doi:10.1371/journal.pone.0239240)

Figure 1A

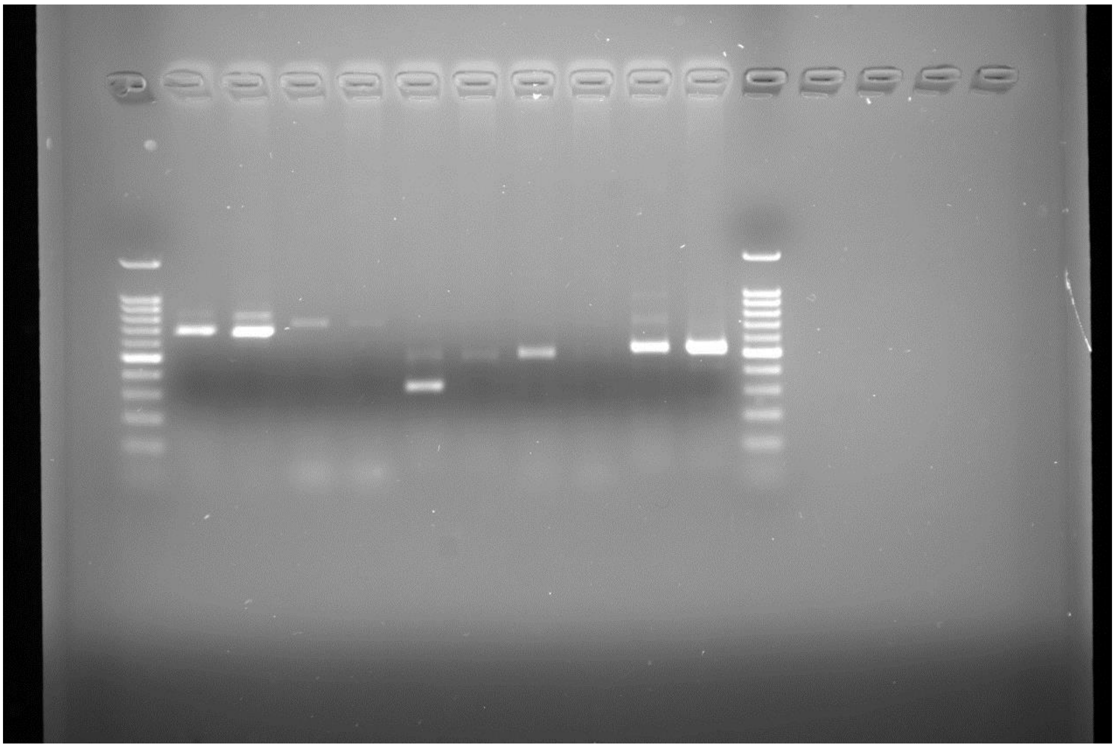

Figure 1B

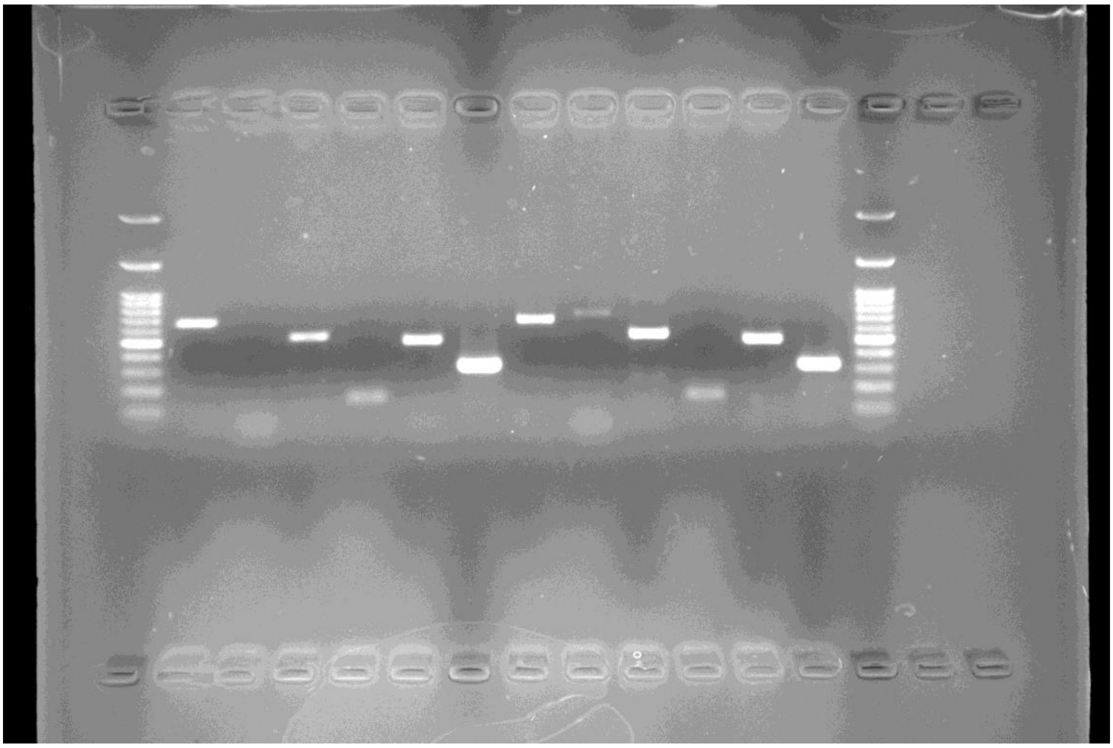

Figure 1C

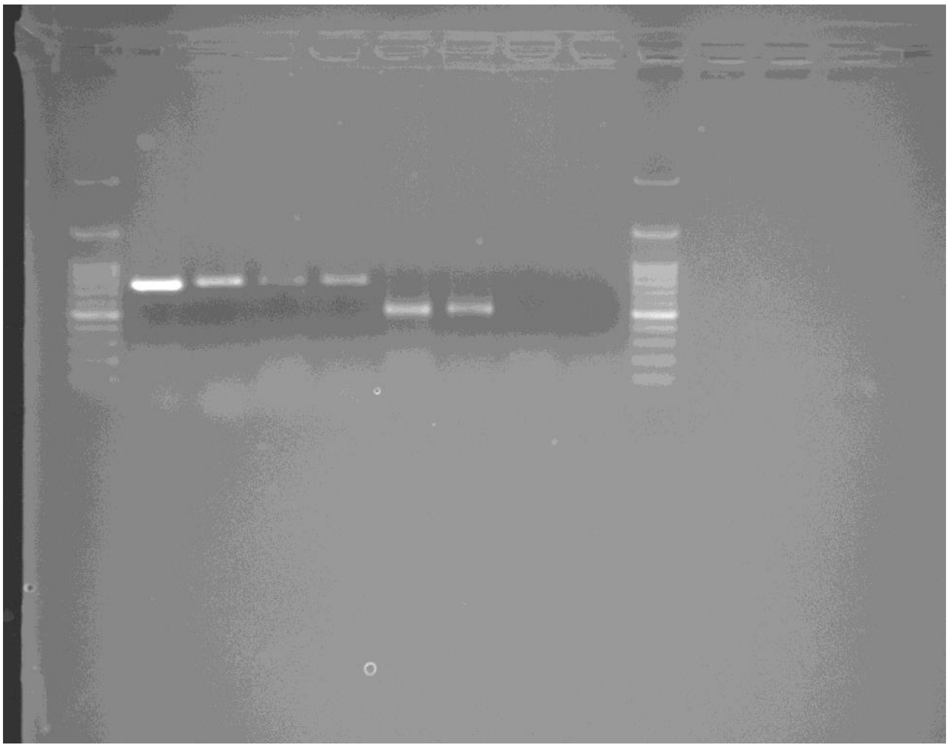

Figure 2D

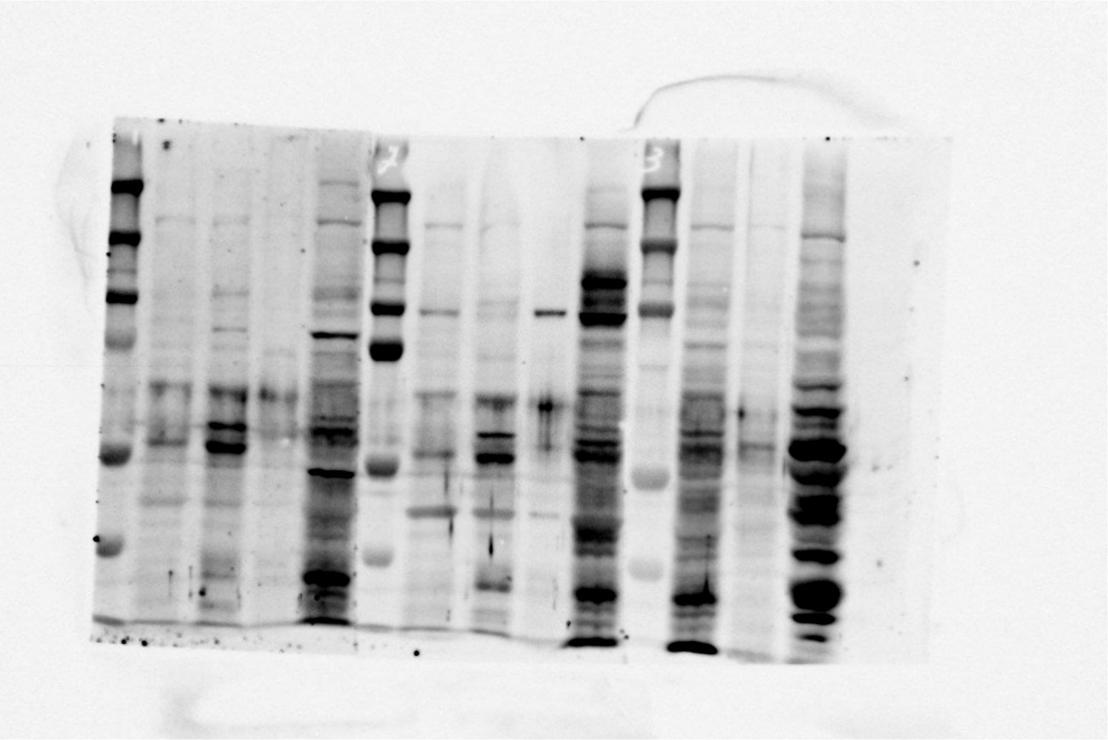

Figure 3A

NHE8 & Angiotensin II -1 hour

GADPH

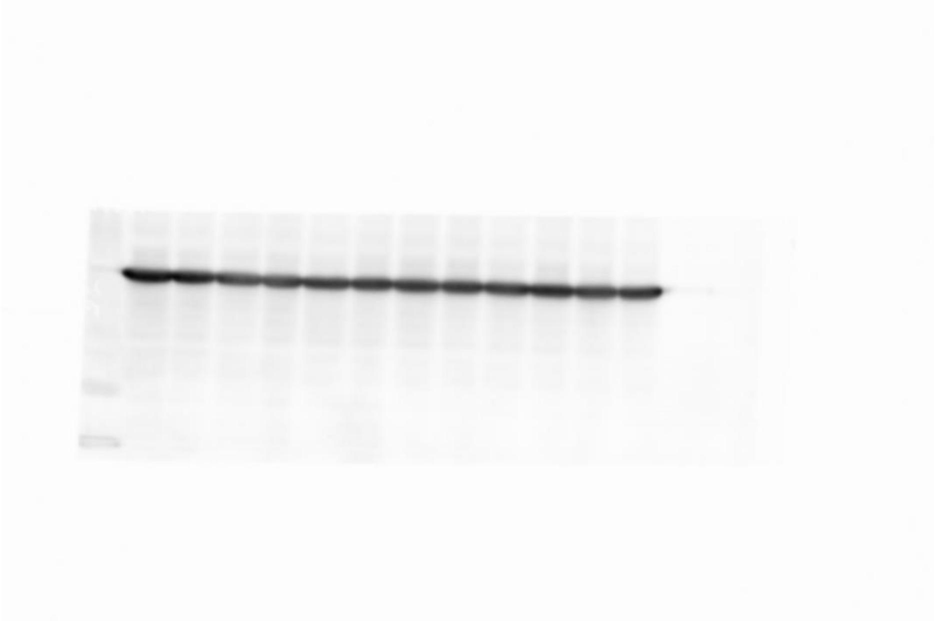

Figure 3B

NHE8 & Angiotensin II -3 hours

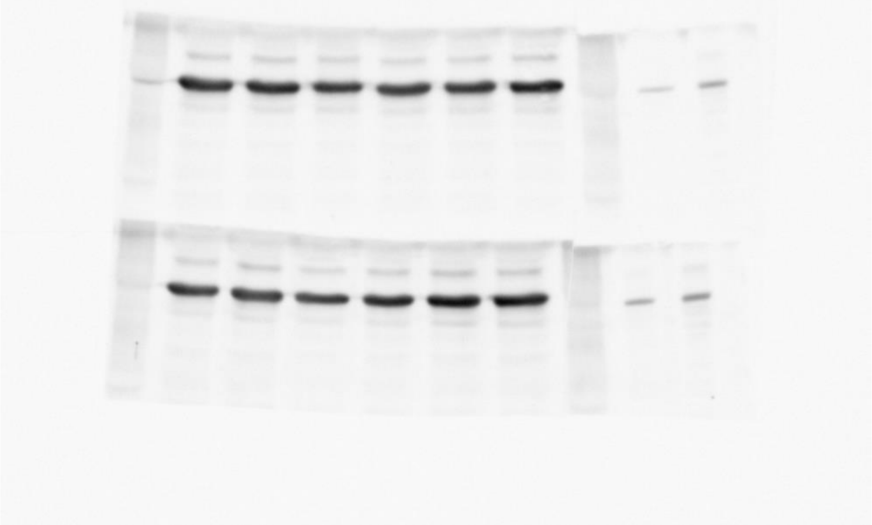

GADPH

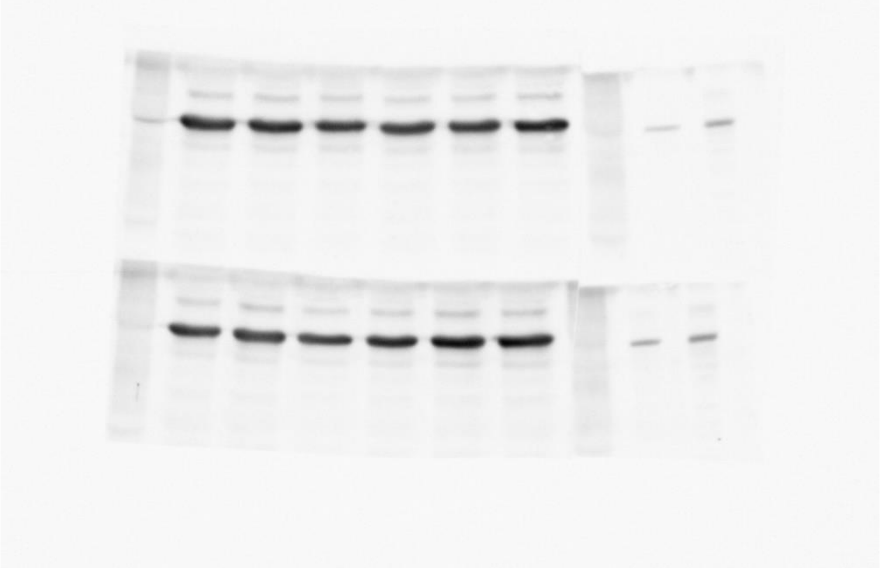

Figures 3C & 3D

NHE8 & Angiotensin II -5 hours

GAPDH

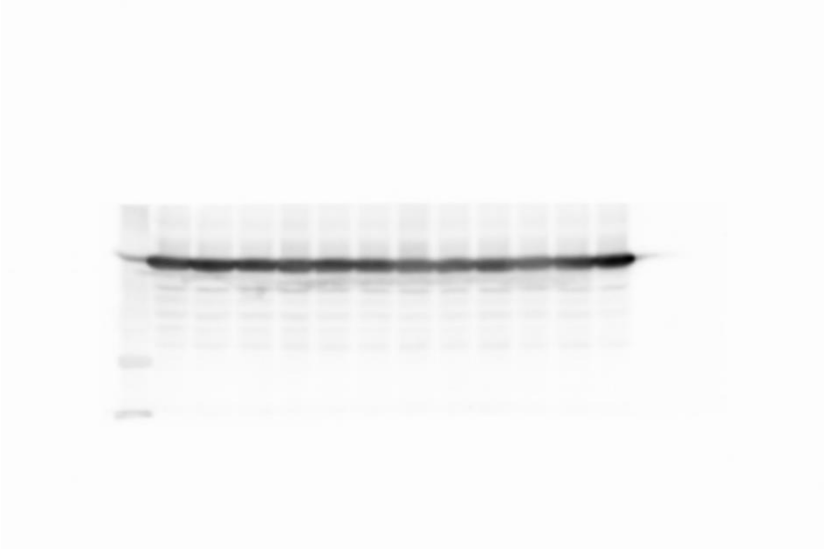

Figure 3E

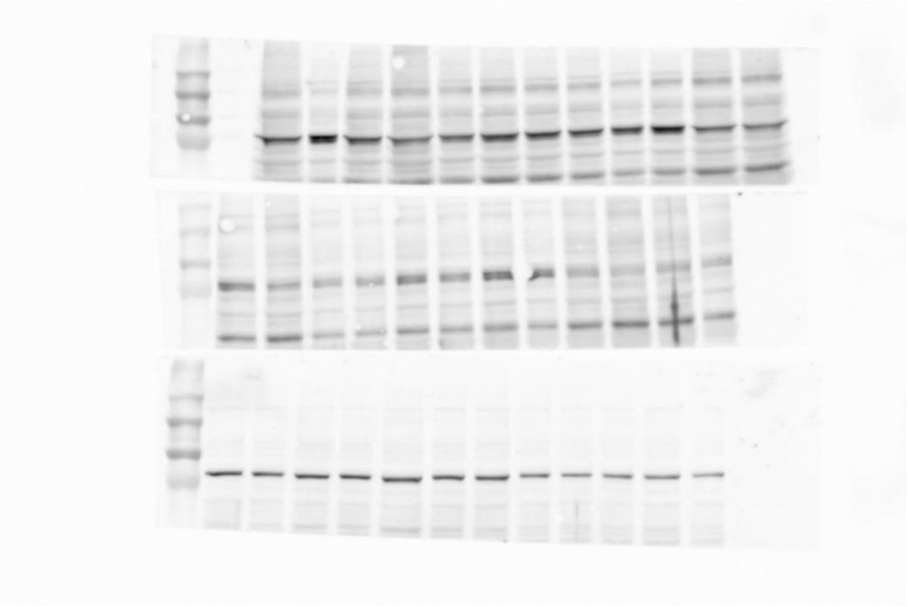

GAPDH

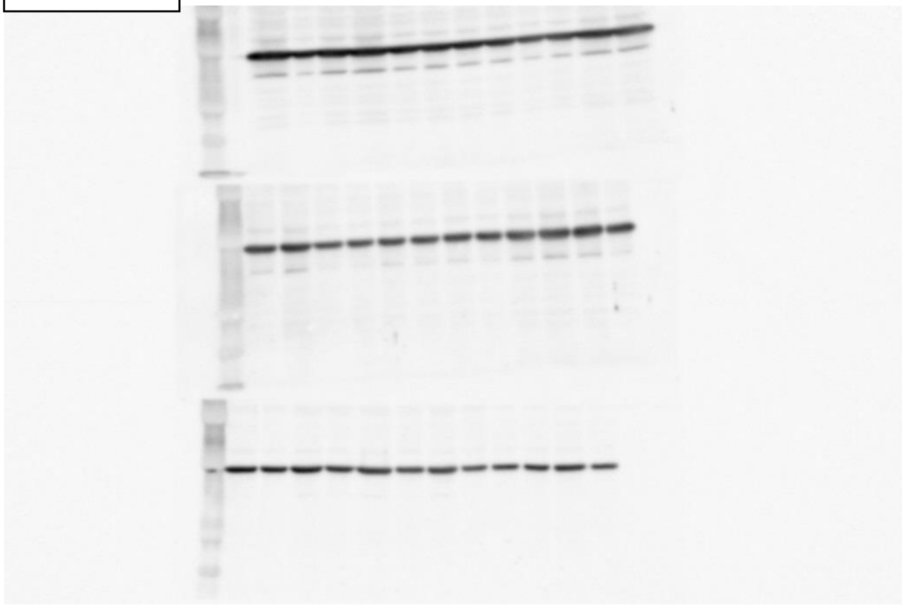

Figure 4A

NHE8 & CHF 1-Week

GAPDH & CHF 1-Week

Figure 4B

NHE8 & CHF 4-Weeks

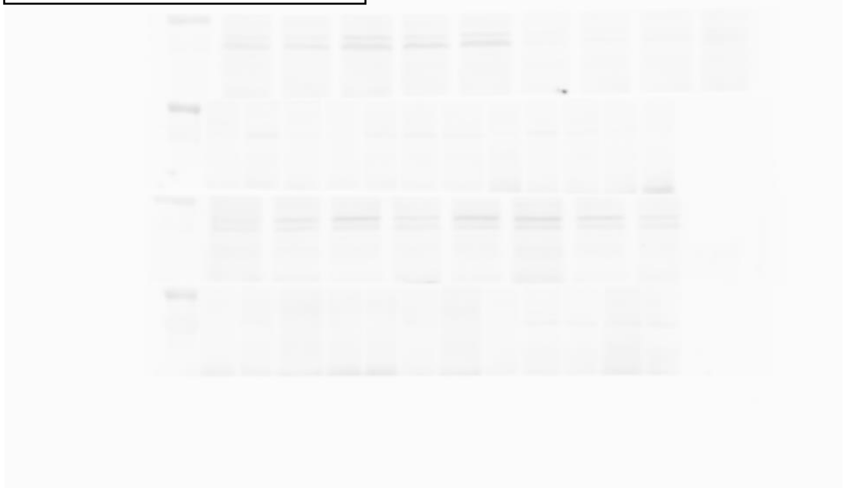

GAPDH & CHF 4-Weeks

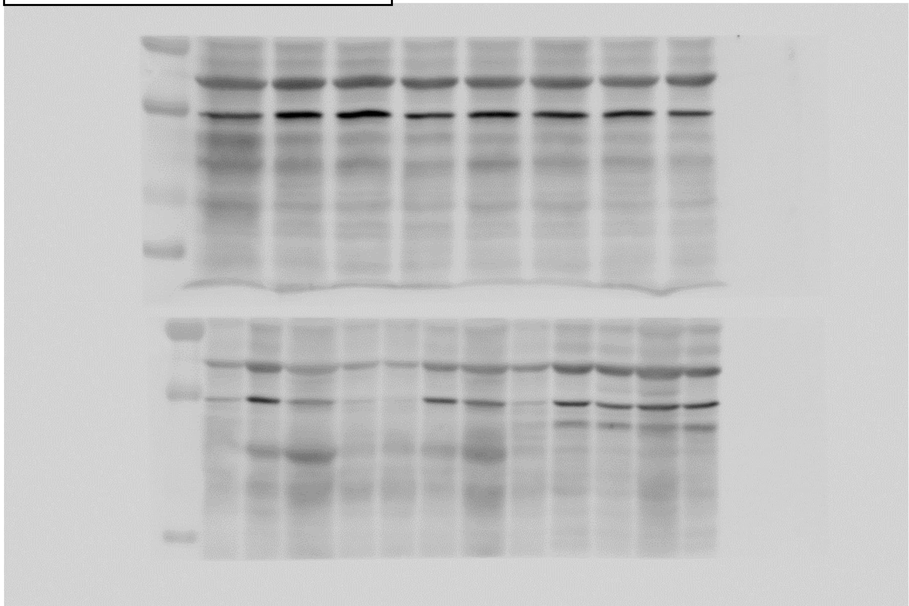

Supplement: S1 Raw images — (PDF) [file pone.0239240.s002.pdf]
